# International Commission on Trichinellosis: Recommendations for genotyping Trichinella muscle stage larvae

**DOI:** 10.1016/j.fawpar.2018.e00033

**Published:** 2019-03-10

**Authors:** Edoardo Pozio, Dante Zarlenga

**Affiliations:** a Department of Infectious Diseases, Istituto Superiore di Sanità, Rome, Italy; b Agricultural Research Service, Animal Parasitic Diseases Lab, Beltsville, MD, USA

**Keywords:** Trichinella, PCR, Genotyping, Diagnosis, Identification

## Abstract

Being able to identify the species or genotype of *Trichinella* is of paramount importance not only for epidemiological studies but to better ascertain the source of outbreaks that still occur worldwide. This has become more critical in recent years given the increase in imported meat products and the relationship that wild animals play in the domestic and sylvatic transmission cycles. In contrast to a time when the genus *Trichinella* was considered monospecific, research in recent years has revealed that the genus consists of 9 species and at least 3 additional genotypes which have yet to be named. Except for a non-encapsulated clade consisting of *Trichinella pseudospiralis*, *Trichinella zimbabwensis*, and *Trichinella papuae*, all members of this genus are morphologically indistinguishable. Thus, identification has been relegated to using PCR and in special cases, DNA sequencing or restriction enzyme digestion. Rather than using a collection of PCR primers specific for each genotype, a single multiplex PCR previously developed for differentiating the major encapsulated and non-encapsulated genotypes has been adopted by the International Commission on Trichinellosis. Since the assay was first developed, other species have been named. Thus, DNA sequencing has been used to validate closely related genotypes. The ICT recommends genotyping be performed as described herein during all outbreaks and whenever *Trichinella* has been found in consumable foods.

## Highlights

• Multiplex PCR of ITS1, ITS2 and ESV rDNA differentiates most *Trichinella* taxa
• Ancillary PCR, RFLP, and sequencing further distinguishes closely related genotypes
• Genotyping should be performed for all food safety-related isolates

## 1 Introduction

The purpose of these recommendations is to present methodology for identifying the species or genotype of *Trichinella* larvae by a multiplex polymerase chain reaction (PCR), or in rare cases, by other PCR-derived methods and DNA sequencing. These techniques can be applied to larvae collected from human biopsies or from muscle tissues of animal origin. In most cases, the multiplex PCR assay (Zarlenga et al., 1999, Zarlenga et al., 2001) can identify pure as well as mixed infections using pooled larvae. Based upon the multiplex PCR for pooled larvae, descriptions of assays using individual worms are also provided (Pozio and La Rosa, 2010) in the event that few worms are obtained. Further, analysis of individual worms may be necessary where mixed infections can cause incorrect diagnoses such as in the delineation of *T. nativa* and *Trichinella-*T6 which are endemic to North America above the −6 °C isotherm. However, the methods described below do not address sampling issues since these will be unique for each purpose and each application. It is advised that the users determine the intent of this methodology prior to sampling and PCR analysis to advance the desired outcome. Data interpretation can be assisted by being familiar with the biogeography and geographical distribution of these genotypes (Karadjian et al., 2017; Pozio and Zarlenga, 2013).

## 2 *Trichinella* morphology

The morphology of adult worms (male, total length 0.62 mm to 1.58 mm, width 25 μm to 33 μm; female, total length 0.952 mm to 3.35 mm, width 26 μm to 43 μm) and of new born larvae (average 110 μm in length, 7 μm in width) does not have any diagnostic importance, since the only stage which can be easily isolated and identified is the muscle larva. Muscle larvae are the infective stage of the parasite, known as first-stage larvae (L1), and subsequent developmental molts occur only after their penetration in the gut mucosa of a new host. The following morphological characters can sexually distinguish male and female muscle larvae. Male larva: total length 0.641 mm to 1.07 mm; width 26 μm to 38 μm; intestinal bulb typically close to the convex surface; in some larvae close to the concave surface; intestine crossing the gonad from the convex to the concave surface; in some larvae, crossing the gonad from the concave to the convex surface and then re-crossing to the concave surface; length of rectum approximately 40 μm to 50 μm. Female larva: total length 0.71 mm to 1.09 mm, width 25 μm to 40 μm; intestinal bulb generally close to the concave surface; intestine on the concave surface; in some larvae, intestine crossing the gonad from the concave to the convex surface and then re-crossing to the concave surface; length of rectum 17 μm to 35 μm; presence of a thickened subcuticular layer in the region of vulva primordium, i.e. on the convex surface at approximately 2/3 of the length along the stichosome. The recovery of nematodes belonging to genera other than *Trichinella* during routine meat inspection suggests that the persons performing the analyses need to be informed of the possibility of false positives and, consequently, the larva morphology should be kept in mind before proceeding with the molecular identification.

## 3 Principle of the methods

The PCR is a molecular biology technique that enables the amplification of specific nucleic acid fragments, of which the initial and terminal nucleotide sequences are known (oligonucleotide pair). If a species (or genotype) has its own characteristic DNA portion, due to its composition and/or dimension, it is possible to choose an oligonucleotide pair enabling its amplification. The PCR amplification is characterized by a high sensitivity and specificity.

A modification of the “conventional PCR” is the multiplex-PCR, where two or more oligonucleotide pairs are used. In this case, it is possible to amplify with a single PCR analysis more than one sequence at the same time.

Currently, 9 sibling species, namely *T. spiralis*, *T. nativa*, *T. britovi*, *T. pseudospiralis*, *T. murrelli*, *T. nelsoni*, *T. papuae*, *T. zimbabwensis*, *T. patagoniensis*, and 3 genotypes, *Trichinella-*T6, *Trichinella-*T8 and *Trichinella-*T9, have been identified in the genus *Trichinella*. The comparative analysis of three nucleotide sequences belonging to the internal transcribed spacers ITS1, ITS2 and expansion segment ESV of the rDNA repeat, allows the unequivocal identification of most epidemiologically relevant taxa: *T. spiralis*, *T. nativa*, *T. patagoniensis*, *T. britovi*, *T. pseudospiralis*, *T. murrelli*, *T. nelsoni*, *T. papuae*, *T. zimbabwensis* and *Trichinella-*T6. Since the size difference between *T. nativa* and *T. patagoniensis* amplicons is of only two base pairs, they can be distinguished by PCR amplification and sequencing of the ESV; *T. britovi* and *Trichinella-*T9 can be distinguished by PCR-RFLP of the CO I mitochondrial gene, and; *T. britovi* and *Trichinella-*T8 can be distinguished by ancillary PCR amplification of the ITS2.

The sizes of the ITS1, ITS2 and ESV fragments produced by the PCR amplification are shown in Table 1.

**Table 1:** Dimension of the expected amplification products (in base pairs) for each taxon.

Locus	*T. spiralis*	*T. nativa*	*T. patagoniensis*	*T. britovi*	*Trichinella* T8	*Trichinella* T9	*T. pseudospiralis*	*T. murrelli*	*Trichinella* T6	*T. nelsoni*	*T. papuae*	*T. zimbabwensis*
ESV	173	129	127	129	129	135	292–360^1^	129	129	155	240	264
ITS1				253	253	253			210			
ITS2								316		404		

1 A multiple band pattern (with 1, 2 or 3 bands) of variable size can be detected in this range.

Using the multiplex-PCR technique with 5 oligonucleotide pairs, it is possible to identify larvae with only one amplification assay to the species or genotype level. Specific instructions are provided in Appendix A.

## Conflict of interest

The authors declare no conflict of interest.

## Figures and Tables

**Table A1:** Set B PCR primer sequences, their codes and amplified target loci.

Primer sequences	Code	Amplified sequence
5′-GTT.CCA.TGT.GAA.CAG.CAG.T-3′ 5′-CGA.AAA.CAT.ACG.ACA.ACT.GC-3′	cp-I·F cp-I.R	ESV
5′-GCT.ACA.TCC.TTT.TGA.TCT.GTT-3′ 5′-AGA.CAC.AAT.ATC.AAC.CAC.AGT.ACA-3′	cp-II·F cp-II.R	ITS1
5′-GCG.GAA.GGA.TCA.TTA.TCG.TGT.A-3′ 5′-TGG.ATT.ACA.AAG.AAA.ACC.ATC.ACT-3′	cp-III·F cp-III.R	ITS1
5′-GTG.AGC.GTA.ATA.AAG.GTG.CAG-3′ 5′-TTC.ATC.ACA.CAT.CTT.CCA.CTA-3′	cp-IV·F cp-IV.R	ITS2
5′-CAA.TTG.AAA.ACC.GCT.TAG.CGT.GTT.T-3′ 5′-TGA.TCT.GAG.GTC.GAC.ATT.TCC-3′	cp-V·F cp-V.R	ITS2

**Table A2:** Single sample Reaction mix: components and volumes.

2× PCR MasterMix	15 μL
Multi-Q-grade H_2_O	4 μL
Set B	1 μL
Total	20 μL

**Table A3:** PCR amplification profile.

Pre-denaturation	4 min/95 °C
Amplification	10 s/95 °C 30 s/55 °C 30 s/72 °C
Number of cycles	35
Final extension	3 min/72 °C

**Table A4:**
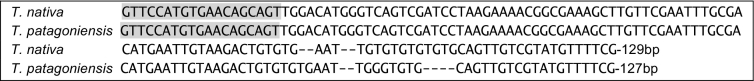
Alignment of the ESV of *T. nativa* and *T. patagoniensis*. Primers are marked in grey.

## Appendix A Methods for genotyping isolates of *Trichinella*

### A.1 Definitions

**CDC**, Centers for Disease Control and Prevention, Office of Health and Safety.

(www.cdc.gov/od/ohs/biosfty/bmbl4/b4af.htm).

**DNA/larva**, DNA extracted from one or more larvae.

**ESV** (Expansion Segment 5), sequence belonging to domain 4 of the nuclear ribosomal gene.

**ITS1** (Internal Transcribed Spacer 1), interspaced sequence 1 of the nuclear ribosomal gene.

**ITS2** (Internal Transcribed Spacer 2), interspaced sequence 2 of the nuclear ribosomal gene.

**MSL**, muscle stage larvae.

**Negative control for the amplification**, reagent grade water; this control is used in the amplification session to verify the efficacy of the multiplex-PCR system.

**Oligonucleotide/Primer**, short sequence (15–30 nucleotide bases) used to amplify a DNA specific fragment.

**PCR**, Polymerase Chain Reaction.

**Positive control for the amplification**, a reference DNA supplied by RLT; this control is used in the amplification session to verify the efficacy of the multiplex-PCR system.

**Positive control for the DNA extraction**, a reference larva analysed in the same working session of test samples, to verify the efficacy of the DNA extraction session.

**Reference larvae**, larvae belonging to a known *Trichinella* species or genotype and with the ISS code (http://www.iss.it/site/Trichinella/index.asp), supplied by the RLT or another reference laboratory

**Reference DNA**, purified DNA belonging to a known *Trichinella* species or genotype with the ISS code (http://www.iss.it/site/Trichinella/index.asp), supplied by RLT or another reference laboratory

**RLT**, Reference Laboratory for Trichinellosis of the World Organization for Animal Health (OIE) and of the International Commission of Trichinellosis (ICT), Rome, Italy (http://www.iss.it/site/Trichinella/index.asp).

**Set B**, mix of 5 oligonucleotide pairs amplifying specific sequences of single species.

**Test sample**, one or more MSL collected from a single infected host and preserved in ethanol, to be identified.

The definitions and terminology used in the UNI EN ISO 22174 standard are applied in the present protocol.

### A.2 Equipment

– Stereo microscope, magnification 60–100×
– Bench top refrigerated centrifuge for 1.5 mL tubes, minimum 15,000 ×*g*
– Freezer −15 °C or colder
– Thermoblock with vibration, temperature range 25–100 °C
– Magnetic separation stand
– PCR thermocycler
– Refrigerator, temperature range +1 to +8 °C
– Horizontal electrophoretic apparatus
– Analytical balance, readability 0 .1g
– UV transilluminator
– Digital imaging system
– Adjustable volume pipettes, volume range: 1–10 μL, 2–20 μL, 20–100 μL, 50–200 μL, 200–1000 μL
– Analytical grade water system production, resistivity ≥18 Ω/cm
– Vortex

### A.3 Reagents and chemicals

The source manufacturers of reagents reported below are for reference purposes only and other suppliers can be used at the discretion of the user but will likely require optimization of the protocol.

**Incubation buffer (IB+)**. Commercial solution: DNA IQ™ System kit, Promega, code DC6701 or DC6700. Once prepared, label the solution with “IB+”. Store per the manufacturer's recommendations.

**Lysis buffer (LB+)** Commercial solution: Tissue and Hair Extraction Kit, Promega, code DC6740. Once prepared, label the solution with “LB+”. Store per the manufacturer's recommendations.

**Paramagnetic resin**. Commercial suspension: DNA IQ™ System kit, Promega, code DC6701 or DC6700. Store per the manufacturer's recommendations.

**Wash buffer**. Commercial solution: Tissue and Hair Extraction Kit, Promega, code DC6740. Once prepared, label the solution with “WB+”. Store per the manufacturer's recommendations.

**Elution buffer**. Commercial solution: Tissue and Hair Extraction Kit, Promega, code DC6740. Store per the manufacturer's recommendations.

 **2× PCR master mix**. 2× commercial solution, Promega, codes: M7501, M7502, M7505 (composition: dATP 400 μM, dCTP 400 μM, dGTP 400 μM, dTTP 400 μM, MgCl_2_ 3 mM, Taq DNA polymerase 50 U/mL); other commercial PCR master mixes should be considered suitable for PCR amplification. Store per the manufacturer's recommendations.

 **PCR primers**. Commercial preparation (Table A1); the lyophilized products are reconstituted with TE 0.1×, according to the manufacturer's recommendations, at a concentration of 100 pmol/μL; the lyophilized product can be stored frozen (−20 °C) for up to 5 years; the reconstituted product can be stored frozen for up to 24 months.

**Set B**. The primer mixture used for the multiplex-PCR is generated by combining an equal volume of the 10 primers; the final concentration corresponds to 10 pmol/μL; 100 μL aliquots are prepared and stored frozen for up to 24 months.

**Loading Buffer (6×)**. Commercial product allowing electrophoresis of amplified DNA to be performed. Store per the manufacturer's recommendations.

**Agarose**. Commercial product suitable for separating DNA fragments <1 kb in size. Store at room temperature for up to 24 months.

**TAE Buffer (50×)**. Commercial product: 2 M Tris-acetate, 50 mM EDTA, pH 8.2–8.4 at 25 °C. Store at room temperature for up to 24 months.

**TAE Buffer (1×)**. 1000 mL preparation: dilute 20 mL of the 50× solution to 1000 mL with water. Store at room temperature for up to 1 month.

**Ethidium bromide solution**. Commercial product: stock 10 mg/L. For the working solution, dilute 1:100,000; for 100 mL solution, add 1.0 μL. Store in the dark at room temperature for up to 24 months. (Note: safer alternatives to ethidium bromide can be used).

Note: ethidium bromide is mutagenic, carcinogenic and teratogenic; wear disposable gloves and handle the solution containing this substance very carefully.

**L50**. Commercial product: DNA molecular weight markers that migrate in increments of 50 bp. Any commercial product of similar size distribution i.e., 50 bp within the 50–500 bp range, can be used. Store refrigerated according to manufacturer's recommendations.

**TE Buffer (1×)**. Commercial product: 10 mM Tris–HCl (pH 8.0), 1 mM EDTA-Na_2_ (pH 7.9–8.1 at 25 °C). Store refrigerated for up to 12 months.

**TE Buffer (0.1×)**. For 100 mL preparation of 0.1× TE, dilute 10 mL of the 1× solution to 100 mL with water. Filter with 0.22 μm filter and store aliquots of 10 mL frozen for up to 24 months.

**Milli-Q grade water**. Resistivity ≥18 MΩ/cm.

**Reference larvae**. *Trichinella* MSL stored in ethanol (95–99%) and with the ISS code supplied by RLT, Rome, Italy (http://www.iss.it/site/Trichinella/index.asp) or another reference laboratory. Reference larvae are supplied after isolation from mouse muscle tissues by HCl-pepsin digestion (see the procedure in OIE 2004). Store frozen for up to 5 years

**Reference DNA**. Genomic DNA purified from reference larvae. Reference DNAs are supplied (1 ng/μL) by RLT. They are produced by RLT from a pool of reference larvae according to the protocols described in Sambrook et al. (1989). Store frozen for up to 5 years

### A.4 Performance of method

#### A.4.1 Sample preparation (pooled or individual larvae)

• For pooled larvae, if the worms are fresh or frozen, one can proceed directly to isolating DNA from MSL. If MSL have been preserved in ethanol, larvae should be washed in water or PBS 3 times prior to DNA isolation.
• For individual larva, ethanol containing MSL is transferred into a petri dish and observed under the stereo microscope. A maximum of 5 MSL are collected and placed in 1.5 mL conical tubes, one MSL in each tube. Excess ethanol is removed and the minimum volume is left.
• Tubes containing larvae are spun at maximum speed for 10 s, the ethanol is removed and the tube is frozen for up to 5 years.

#### A.4.2 DNA isolation

##### A.4.2.1 DNA extraction from pooled larvae

• Total nucleic acids can be isolated from a preparation of mixed MSL by any one of a multitude of methods for isolating genomic DNA from tissues including conventional proteinase K:SDS digestion followed by organic extraction. Numerous kits are commercially available for this purpose; however, isolation should involve the use of proteinase K to digest through the parasite cuticle. These procedures need not require removal of RNA; the presence of total RNA during PCR will not affect PCR amplification.

##### A.4.2.2 DNA extraction from individual larva

• If not otherwise specified, the procedure is carried out at room temperature.
• Each working session requires that a reference larva be submitted to the DNA extraction procedure and identified as “positive control for the extraction”; inclusion of a negative control consisting of extraction reagents only can also be considered to control for cross-contamination during the extraction procedure.
• Before beginning, prepare sufficient volumes of the IB+ and LB+ solutions

###### A.4.2.2.1 Procedure

– Centrifuge the tubes containing the MSL to be identified at maximum speed (>15,000 g) for 10 s.
– Add 20 μL of IB+.
– Incubate at 55 °C for 30–60 min in the thermoblock. During incubation, shake at 1400 vibrations/min.
– Centrifuge, as above.
– Add 40 μL of LB+.
– Add 4 μL of paramagnetic resin. Be sure to completely suspend resin by vortexing before dispensing.
– Incubate for 5–10 min at 25 °C in the thermoblock. During incubation, shake at 1400 vibrations/min.
– Place the tubes in the magnetic separation stand and wait for 30–60 s, so that the paramagnetic resin particles are blocked by the magnetic field on the tube wall.
– Discard the liquid phase by aspirating; avoid dislodging the resin particles from the sides of the tube.
– Add 100 μL of LB+ and suspend the resin particles by vortexing.
– Place the tubes in the magnetic separation stand.
– Discard the liquid phase by aspirating.
– Washing: add 100 μL of WB+ 1× and suspend the resin particles by vortexing.
– Place the tubes in the magnetic separation stand.
– Discard the liquid phase by aspirating.
– Repeat the washing step 3 times.
– After the last wash, leave the tubes open and allow the resin particles to air dry for 15–20 min.
– Add 40 μL of the elution buffer and gently suspend the resin particles, do not vortex.
– Incubate at 65 °C for 5 min. During incubation, shake at 1400 vibrations/min.
– Place the tubes in the magnetic separation stand. Collect 30 μL of the liquid phase and transfer it to a 1.5 mL tube.

The resulting extract is defined as “larval DNA” and should be stored frozen. Under these conditions, it can be stored for up to 5 years.

#### A.4.3 PCR amplification

• Unless otherwise indicated, store tubes on ice; use pipette tips with barrier and wear disposable gloves.
• At each working session, use a positive and a negative amplification control. Use reference DNA as positive control and Multi-Q-grade water as negative control.
• For DNA isolated from a mixed population of larvae, PCR amplification can be performed as described below; however, the amount of DNA to be tested must be determined empirically beginning with dilutions of 1:200 of stock isolated MSL DNA and adjusting the volume of water accordingly in the final Reaction mix.

##### A.4.3.1 Procedure

– Thaw DNA/larva, 2× PCR MasterMix, Set B, and positive amplification controls.
– Mark with a progressive number an adequate number of 200 μL PCR tubes.
– Prepare an adequate cumulative volume of the Reaction mix. Evaluate the volume based on a single sample of Reaction mix (Table A2) and of the total number of samples plus two (1 for the positive amplification control and 1 for the negative one).

– Vortex the Reaction mix and centrifuge at maximum speed for 10 s.
– Transfer 20 μL of the Reaction mix to each PCR tube.
– Add 10 μL of the larval DNA to be tested to each tube.
– Close the tubes, mix by vortexing and centrifuge at maximum speed for 10 s.
– Start the PCR amplification (Table A3) on the thermocycler device; wait for the temperature to reach 95 °C and insert the tubes in the thermoblock after pausing the instrument.

– At the end of the PCR amplification, centrifuge the tubes at maximum speed for a few seconds.
– Add 5.0 μL of loading buffer (6×).
– Vortex and centrifuge the tubes at maximum speed for a few seconds.
– Keep tubes on ice or refrigerate until ready for gel electrophoresis.

#### A.4.4 Gel electrophoresis

##### A.4.4.1 Procedure

– Assemble the electrophoresis apparatus per the manufacturer's recommendations. For the gel preparation, use a comb suited for the number of samples.
– Add 2 g agarose suitable for separating small fragments in 100 mL TAE Buffer (1×) in a glass beaker.
– Gently suspend the powder by agitation.
– Boil the agarose suspension for 30 s. If the agarose is not completely dissolved, continue to boil for another 30 s. Repeat as necessary until the solution is homogeneous.
– QT electrophoresis solution to 100 mL with water.
– Allow the agarose solution to cool.
– Before it solidifies (at about 47 °C), add 1.0 μL of ethidium bromide solution.
– Swirl gently to uniformly dissolve the ethidium bromide and pour the agarose in the gel tray previously prepared.
– Wait for the gel to solidify, which requires at least 30 min.
– Place the tray with the gel in the electrophoresis apparatus.
– Cover the gel with TAE Buffer (1×) and gently pull out the comb.
– The first and last wells are loaded with 15 μL of the L50 solution.
– Load in each well, 20 μL of the PCR product giving attention to the progressive numbering of the tubes.
– Connect the electrophoresis apparatus with the power supply and set to 10 v/cm of gel.
– Run the gel for about 30 min or until the fastest dye, contained in the loading buffer, reaches 1 cm from the gel border.
– After 30 min, switch off the power supply, place the gel under UV illumination and check the band separation. The electrophoresis run is adequate if one can easily distinguish all bands of the molecular weight marker ranging from 50 to 500 bp. If the separation is incomplete, continue the run.
– At the end of the run, transfer the gel to the imaging system and print the result.

### A.5 Data interpretation

• The size of the PCR bands revealed by the electrophoresis is evaluated by comparing to the reference molecular weight standards L50. Visual evaluation is adequate in most cases since the differences among species are macroscopic (see Table 1 of Recommendations).
• The amplification result is considered valid if:
  a) the positive DNA control shows an amplification product in Table 1;
  b) the negative control does not show an amplification product higher than a primer dimer i.e. 75 bp
  c) the positive extraction product control shows an amplification product as in Table 1;
• Species identification is made by comparing the size of the band(s) produced by the unknown sample(s) with those shown in Table 1. Results are interpreted as follows:
– If the amplification band is of 173 bp, the larva is identified as belonging to *T. spiralis*.
– If the amplification band is of 129 bp, the larva is identified as belonging to *T. nativa*.
– If the amplification band is of 127 bp, the larva is identified as belonging to *T. patagoniensis*.
– If the amplification bands are of 129 bp and 253 bp, the larva is identified as belonging to *T. britovi* or *Trichinella*-T8.
– If the amplification bands are of 135 bp and 253 bp, the larva is identified as belonging to *Trichinella*-T9.
– If the amplification band pattern (1, 2 or 3 bands) is in the range between 292 and 360 bp, the larva is identified as belonging to *T. pseudospiralis*.
– If the amplification bands are of 129 bp and 316 bp, the larva is identified as belonging to *T. murrelli*.
– If the amplification bands are of 129 bp and 210 bp, the larva is identified as belonging to *Trichinella*-T6.
– If the amplification bands are of 155 bp and 404 bp, the larva is identified as belonging to *T. nelsoni*.
– If the amplification band is of 240 bp, the larva is identified as belonging to *T. papuae*.
– If the amplification band is of 264 bp, the larva is identified as belonging to *T. zimbabwensis*.
– If the result is deemed valid but the sample shows an unexpected band of a size not reported in Table 1, the identification is defined as “unknown”.

Whenever possible, five larvae should be tested for each test sample. The isolate identification is considered valid if at least one larva can be identified. In the case of an invalid result, an additional 5 larvae should be tested.

As reported above, *T. murrelli* shows two bands of 129 bp and 316 bp. However, a third weaker band of 256 bp can occur. This band can lead to misidentification, since it is similar to the 253 bp band displayed by *T. britovi*.

After multiplex PCR, to distinguish between *T. nativa* and *T. patagoniensis* and between *T. britovi*, *Trichinella*-T8 and *Trichinella*-T9, only a high-resolution device for analysing amplification fragmentation patterns of closely migrating products (e.g., capillary electrophoresis apparatus; high resolution agarose gel; acrylamide gel) will unequivocally differentiate these taxa (except for *T. britovi* and *Trichinella*-T8). If such a device is not available and PCR amplicons can be separated only by 2% agarose gel, the following three molecular methods (Sections A.6, A.7 and A.8) will permit unequivocal identification of these taxa.

### A.6 PCR and sequencing of the ESV to distinguish between *T. nativa* and *T. patagoniensis*

• When using multiplex-PCR, larvae belonging to *T. nativa* display a band pattern very similar (only two base pairs of difference, 129 bp versus 127 bp) to the band pattern of *T. patagoniensis* larvae. These two species can be distinguished by the amplification with the primer set cp-I·F and cp-I.R, and sequencing of the ESV. *T. nativa* yields a fragment of 129 bp, whereas *T. patagoniensis* yields a fragment of 127 bp. The alignment is shown in Table A4. Based on current information, *T. patagoniensis* is circulating only in South America. For this reason, it is suggested that this method be used only if the MSL with a *T. nativa* gel banding pattern by multiplex-PCR originated from this continent.

### A.7 PCR-RLFP to distinguish between *T. britovi* and *Trichinella*-T9

• When using multiplex-PCR, larvae belonging to the T9 genotype display the same band pattern as *T. britovi*. These two taxa can be distinguished by PCR-RFLP based on the CO I mitochondrial gene sequence. According to the available information, the T9 genotype is circulating only in Japan; for this reason, it is suggested that this method be used only if the MSL with a *T. britovi* band pattern by multiplex-PCR originated from this country.
• Reagents: (i) 2× PCR Master Mix; (ii) oligonucleotides for CO I, as target locus: L6625, F 5′-TTYTGGTTYTTCGGKCACCC-3′; H7005, R 5′-ACGACGTAGTAGGTRTCRTG-3′; (iii) reference DNA from *T. britovi* and *Trichinella*-T9 (ITRC, Rome, Italy); and (iv) purified DNA of the larvae to be tested.

#### A.7.1 Procedure

This procedure consists of two steps: (1) PCR amplification of samples; and (2) restriction enzyme digestion of the PCR products.

(1) PCR amplification: The total volume of the amplification reaction is 50 μL.

– The amplification solution is prepared by adding sequentially: 25 μL 2× PCR Master Mix, 13 μL water, 1 μL of each primer, and 10 μL of DNA extract from the single larvae to be tested;
– The amplification cycle, preceded by a pre-denaturation step of 4 min/94 °C, is: 60 s/94 °C, 60 s/48 °C, 60 s/72 °C, for 35 cycles, followed by a final extension step of 3 min/72 °C. It is recommended that a hot start Taq DNA polymerase be used instead of a standard enzyme;
– At the end of the amplification, centrifuge the tubes at maximum speed for a few seconds;
– Add the DNA loading buffer and load the samples onto a 2% agarose gel to view the result (both *T. britovi* and *Trichinella* T9 display a 419 bp band). The amount of DNA product present in each sample should be evaluated by eye; and
– Store the amplified products at −20 °C until use.

(2) Restriction analysis; combine all reagents while keeping tubes on ice.

– For each sample, transfer at least 100 ng of the amplified product into a 1.5 mL reaction tube;
– Set the volume to 10 μL with water;
– Add to the reaction tube: 5 μL of 10× *Mse*I restriction enzyme reaction buffer, 1 μL of *Mse*I (10 U/μL) restriction enzyme, and water up to 50 μL. When working with multiple samples, a quantity of amplification solution that is sufficient for all the samples can be prepared all at once; aliquot the solution in 1.5 mL tubes containing the PCR products to be digested;
– Mix and incubate at 37 °C for 60 min;
– Stop the reaction by heating at 65 °C for 20 min and then spinning for a few seconds;
– Add the DNA loading buffer, visualize the results by 2% agarose gel electrophoresis as described for PCR;
– Evaluate the results per the expected size pattern: for *T. britovi* five bands of 22, 62, 64, 70, and 201 bp; for *Trichinella* T9, two bands of 92 and 327 bp.

### A.8 PCR to distinguish between *T. britovi* and *Trichinella*-T8

• By multiplex-PCR, the larvae of *Trichinella* T8 display the same band pattern as *T. britovi* larvae. These two taxa can be distinguished by PCR based on a 21 bp deletion in the ITS2 sequence of *Trichinella* T8. According to the available information, the T8 genotype is circulating only in sub-Saharan Africa; it is thus recommended that this method only be used if the larvae with a *T. britovi* band pattern by multiplex-PCR originate from this region.
• Reagents: (i) oligonucleotides: ITS2, as target: ITS2G.F 5′-CCGGTGAGCGTAATAAAG-3′, ITS2G.R, 5′-TACACACAACGCAACGAT-3′; (ii) reference DNA from *T. britovi* and from *Trichinella* T8 (ITRC, Rome, Italy); and (iii) purified DNA of *Trichinella* larvae to be tested.

#### A.8.1 Procedure

– The amplification solution (30 μL) is prepared by adding sequentially: 15 μL 2× PCR Master Mix, 3 μL water, 1 μL of each primer and 10 μL of DNA extract from a single MSL or control DNA. When working with multiple samples, a quantity of amplification solution that is sufficient for all of the samples can be prepared all at once; aliquot the solution into the PCR tubes containing the DNA samples to be tested;
– Close the tubes, mix by a vortexing, and centrifuge at maximum speed for a few seconds;
– The amplification cycle, preceded by a pre-denaturation step of 4 min/94 °C, is: 30 s/94 °C, 30 s/51 °C, 60 s/72 °C, for 35 cycles, followed by a final extension step of 3 min/72 °C. It is recommended that a hot start Taq DNA polymerase be used instead of a standard enzyme;
– At the end of the amplification, centrifuge the tubes at maximum speed for a few seconds;
– Add the DNA loading buffer, and visualize the results by 2% agarose gel electrophoresis;
– Evaluate the result according to the expected size pattern: *T. britovi*, band of 125 bp; *Trichinella*-T8, band of 104 bp.

### A.9 Safety measures

This method should be carried out only by authorized personnel. The operator should wear appropriate personal protective equipment during the test performance. For general safety measures, refer to the guidelines of CDC.

### A.10 General references

Karadjian, G., Heckmann, A., Rosa, G.L., Pozio, E., Boireau, P., Vallée, I. 2017. Molecular identification of *Trichinella* species by multiplex PCR: new insight for *Trichinella murrelli*. Parasite 24, 52. doi: https://doi.org/10.1051/parasite/2017053.

OIE Manual of Diagnostic Tests and Vaccines for Terrestrial Animals, 2004, V Edition, pp. 380–386.

Pozio, E., La Rosa, G. 2010. *Trichinella*. Liu D. (Ed.), Molecular detection of foodborne pathogens. CRC Press Taylor and Francis Group, Boca Raton, London, New York, pp. 851–863.

Pozio, E., Zarlenga, D.S. 2013. New pieces of the *Trichinella* puzzle. Int. J. Parasitol. 43, 983–997.

Sambrook J, Fritsch EF, Maniatis T. 1989. Molecular Cloning: A Laboratory Manual. Cold Spring Harbor Laboratory Press, NY, Vols. 1, 2, 3.

UNI EN ISO 22174: 2005. Microbiologia di alimenti e mangimi per animali – reazione a *catena* di polimerizzazione (PCR) per la ricerca di microrganismi patogeni negli alimenti – requisiti generali e definizioni.

Zarlenga, D.S., Chute, M.B., Martin, A., Kapel, C.M. 1999. A multiplex PCR for unequivocal differentiation of all encapsulated and non-encapsulated genotypes of *Trichinella*. Int. J. Parasitol. 29, 1859–1867.

Zarlenga, D.S., Chute, M.B., Martin, A., Kapel, C.M. 2001. A single, multiplex PCR for differentiating all species of *Trichinella*. Parasite 8, S24–S26.
